# The missing data problem in population genomics and statistical methods to address them

**DOI:** 10.1093/g3journal/jkaf269

**Published:** 2026-01-07

**Authors:** Arun Sethuraman

**Affiliations:** Department of Biology, San Diego State University, San Diego, CA 92182, United States

**Keywords:** genomics, population genetics, missing data

The “Missing Data” problem is prevalent across all statistical inference, owing to the “absence of some part of a familiar data structure” (Efron 1994). Population genomic datasets are riddled with missing data (Fig. 1)—broadly classified as data missing at random (e.g. due to degradation, sequencing errors), data missing “on purpose” (e.g. due to sequencing strategies like genotyping by sequencing), and data missing due to unknown evolutionary history (e.g. introgression from ancestral ghost populations). Editors and scientific contributors to both the GSA's journals, Genetics and G3 have continually highlighted statistical issues and pitfalls with inference in the presence of missing data (McIntyre 2025), particularly in an age of Biobank scale population genomic datasets. Here I highlight studies, including those that have been recently published in Genetics and G3 towards systematically assessing the effects of missing data problems and addressing them towards inference in a variety of population genomics questions.

## The missing data problems

Genotyping and sequencing errors might lead to incorrectly called nucleotide bases (Nielsen and Signorovitch 2003). These erroneous base calls are often either discarded or imputed with respect to a reference genome but could lead to ascertainment biases in calling variant sites and potentially incorrect inferences. Ascertainment biases during variant discovery, mapping biases during read alignment, and imputation biases during missing data inference are also exacerbated in ancient genomic datasets, which suffer from systematic degradation from DNA damage, and cytosine deamination (described by Günther et al. 2025).

A second type of missing population genomic data arises due to molecular methodological issues with genotyping or sequencing. This is particularly exacerbated in methods that utilize reduced representation genomic sequencing, often called Genotyping By Sequencing (GBS) methods such those using SNP-chips, which suffer from ascertainment bias. For instance, Gómez-Palacio et al. 2024 in a recent issue of G3 discuss the “robustness’ of demographic inference from SNP chip vs whole genome sequencing (WGS) data in *Aedes aegypti*, highlighting the utility of SNP-chips as a lower cost alternative to WGS in non-model systems. Other lower coverage approaches such as Ultra-Conserved Element-based sequencing (Portik and Wiens 2021), PoolSeq (Kofler et al. 2011; Carvalho et al. 2023), and Restriction Site Associated DNA sequencing (RADseq—Baird et al. 2008) remain powerful low-cost strategies for phylogenetic inference as described in several recent studies in G3 (e.g. Forest et al. 2024 in woodpeckers, Parys et al. 2025 in bees), variant calling (Chen et al. 2025), identifying meiotic drive loci (Barbash et al. 2023), and in inference of evolutionary history, despite missing data issues. These issues arise because of allele dropout due to un/under-sequenced genomic regions, along with polymorphisms present across restriction sites, being absent in subsequent sequencing output (Andrews et al. 2016). Allele dropout introduces systematic biases and limitations (Fu 2014) while using RADseq data to perform inference of evolutionary history (Nunziata and Weisrock 2018).

Thirdly, genomic data could also be missing due to the presence of unsampled genomic variation from populations that are either extinct, severely depleted, or impossible to sample. Termed “ghost” populations, ancestral or continuing gene flow from these “ghosts” leaves signatures on extant and/or sampled genomes that often go undetected. “Ghost” variation in sampled genomes also introduces biases in estimates of evolutionary history, if unaccounted for (Beerli 2004; Slatkin 2005; Tricou et al. 2022; Sethuraman et al. 2025).

## Solving missing data problems

There are three common approaches to handling missing data in genomics that are widely implemented in popularly used tools in the field of population genomics, several of which are described in Genetics and G3—(1) missing data is considered as data missing at random (e.g. popular tools like STRUCTURE—Pritchard et al. 2000, ADMIXTURE—Alexander et al. 2009), and therefore not accounting for in the inference model, (2) imputing missing data—i.e. filling in missing data with respect to a reference genome or panel (e.g. see a detailed treatment of the issue of “switch-errors” during local ancestry inference in Avadhanam and Williams 2025, or while imputing ancient DNA data in Ausmees et al. 2022), and (3) mean imputation—i.e. filling in missing data with a mean population statistic (e.g. DAPC/ADEGENET—Jombart 2008). Nonetheless, all three approaches offer little improvement on the performance of model-based inference methods (Le Morvan and Varoquax 2024) and can bias non-model-based inferences (e.g. PCA—see Yi and Latch 2022).

A few researchers have developed corrected method-of-moments estimators of population genetic summary statistics, such as allele frequencies and ancestry proportions (Günther et al. 2025), effective population size (Ne—Peel et al. 2013), Watterson's θ, Tajima's D (Bailey et al. 2025), Fay and Wu's H (Ferretti et al. 2012), nucleotide diversity (*π*—Korunes and Samuk 2021) that account for genotyping errors. Others have developed methods that include missing genotypic data in the inference model, specifically while inferring haplotypic phase (e.g. Montero-Tena et al. 2024), effective population size (e.g. Hong et al. 2024) in genome-wide association studies (e.g. Jørsboe and Albrechtsen 2022), and ancestry estimation (e.g. Vitale et al. 2025).

**Fig. 1.**
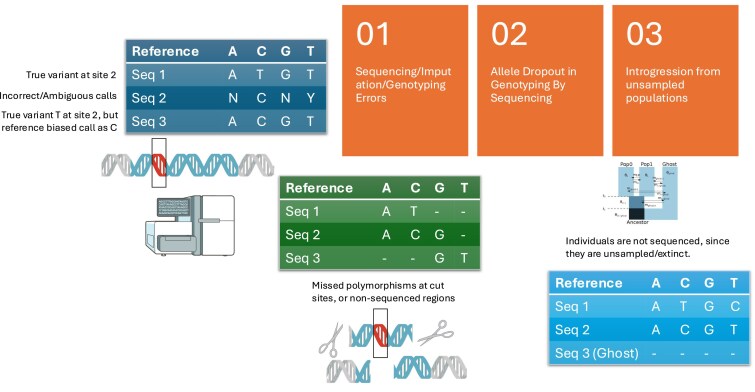
Infographic showing three types of missing data problems in population genomics, concerning (1) sequencing/imputation/genotyping errors, (2) allele dropout in genotyping by sequencing, and (3) missing data due to introgression from unsampled populations.

Recently, we (Sethuraman et al. 2025) present an account of systematic biases in the estimation of evolutionary history while not accounting for the presence of gene flow from unsampled ghost populations; we developed theoretical expectations for estimates of commonly utilized population genomic summary statistics such as nucleotide diversity, genetic differentiation, and Tajima's D, while applying extensive simulations to discuss how increased degree of ancestral gene flow from unsampled ghosts will bias inference from sampled extant populations. We are continuing to develop methods to address these biases in estimation of evolutionary history while making evolutionary inferences.

Going forward, I see two important venues for the population genomics community to undertake.

1. Developing simulators of missingness: Simulators of population-scale genomic data come in two flavors—backward-time, or coalescent simulators, such as *msprime* (Baumdicker et al. 2022) and *fsc28* (Excoffier et al. 2021), and forward-time simulators, such as *SLiM* (Haller and Messer 2019). Both flavors have been used extensively in fundamental studies of evolutionary population genomics, especially to create “null” and “alternate” hypotheses for testing (a) validity of new evolutionary inference methods, and (b) establishing expectations under neutral/selective evolutionary processes. However, in reality, data generated using genomic sequencing platforms are replete with missing data of varying types, that are not addressed in simulators. Therefore, to address this important gap in simulations, we need to develop missingness simulators in population genomics that can account for sequencing/genotyping errors and in restriction enzyme associated sequencing methods (RADseq). For instance, the *RADinitio* method of Rivera-Colón et al. 2021 describes a new simulator for systematic processing of forward-time simulations via SLiM to include missingness. Thereon, we should be characterizing the effects of missingness and imputation in estimation of evolutionary history via systematic assessments of simulated and empirical datasets to understand the effects of missingness in large population genomic datasets under a variety of evolutionary demographic models. Another method, developed by Goulart and Samuk (2025) utilizes the *msprime* pipeline to accurately introduce missing data across various ploidy levels.
2. Develop statistical methods to include missing data in the inference: Current methods for estimating evolutionary history from population-scale genomic data are yet simplistic, and for the most part, fail to account for the presence of missing data. As elegantly discussed by Nielsen and Signorovitch 2003, we require new methods that will incorporate different kinds of missingness in the generative models—I hope that this editorial will be an invitation for researchers to consider the effects of missingness while developing such methods.
